# Postmortem Genetic Testing in Sudden Unexpected Death: A Narrative Review

**DOI:** 10.7759/cureus.33728

**Published:** 2023-01-13

**Authors:** Shahad A Alzahrani, Nour F Alswaimil, Alia M Alammari, Wala H Al Saeed, Ritesh G Menezes

**Affiliations:** 1 Department of General Medicine, College of Medicine, Imam Abdulrahman Bin Faisal University, Dammam, SAU; 2 Department of Pathology, College of Medicine, Imam Abdulrahman Bin Faisal University, Dammam, SAU

**Keywords:** molecular genetics, molecular autopsy, genetic testing, postmortem, sudden death

## Abstract

Sudden unexpected death (SUD) is one of the challenging situations encountered in forensic medicine. As a rule, a comprehensive forensic assessment is performed to identify the cause of death in such cases; however, the absence of findings suggestive of a cause, i.e., a negative autopsy, warrants further investigation such as a molecular autopsy. In this review, we aim to highlight the genetic causes of SUD, tools used in a molecular autopsy, and the role of screening in surviving relatives. As per several guidelines, the most preferred samples for DNA extraction are whole blood and fresh frozen tissues. Furthermore, Sanger sequencing and next-generation sequencing are the technologies that are used for genetic analysis; the latter overcomes the former’s drawbacks in terms of cost-effectiveness, time consumption, and the ability to sequence the whole exome. SUD have diverse etiologies; we can generally classify them into cardiac and non-cardiac causes. Regarding cardiac causes, many conditions having an underlying genetic basis are included, such as channelopathies and cardiomyopathies. Regarding non-cardiac causes of SUD, the main etiologies are epilepsy and metabolic disorders. Nevertheless, it has been proposed that there is a genetic overlap between channelopathies, especially long QT syndromes and epilepsy. Additionally, fatty acid oxidation disorders are major metabolic conditions that are caused by certain genetic mutations that can lead to SUD in infancy. Since many SUD causes have an underlying genetic mutation, it is important to understand the genetic variations not only to recognize the cause of death but also to undertake further preventive measures for surviving relatives. In conclusion, a molecular autopsy has a major role in the forensic examination of cases of SUD.

## Introduction and background

Sudden unexpected death (SUD), at any age, is a catastrophic event that results in a significant impact on the grieving family. In the literature, there is considerable variation regarding the definition of the term SUD. According to the International Classification of Diseases, 11th Revision (ICD-11) by the World Health Organization, SUD (or sudden death, cause unknown) is defined as instantaneous death, or a fatal event that is neither traumatic nor explained by a disease, which occurs within 24 hours of symptoms’ onset in an apparently healthy individual [1]. Sudden death could be cardiac, non-cardiac, or unexplained even after complete forensic investigations. If the cause of death is identified to be a disease of the heart or a vascular anomaly in the absence of extra-cardiac etiology at postmortem examination, it is called sudden cardiac death (SCD) [2]. SUD can tragically occur in all age groups: infants, children, young adults, and the elderly. Generally, “sudden death in the young” is a term that includes victims between the age of 1-40 years, whereas sudden unexpected death in infancy (SUDI) is the equivalent term used for those younger than the age of one year [2,3]. In all conditions, SUD results in considerable adverse psychological consequences, fear, and unanswered questions in surviving relatives.

SUD is one of the most challenging scenarios encountered by forensic pathologists. It is mandatory to perform an autopsy in all cases of SUD in many jurisdictions. A detailed and comprehensive forensic evaluation should be performed aiming at identifying the cause of death and assisting law enforcement agencies in resolving any suspicions regarding death. Generally, this includes obtaining the medical history, external examination, and gross and microscopic autopsy, followed by advanced forensic investigations such as toxicological analysis, postmortem imaging, and genetic testing [4-6].

In about 65-85% of cases, a macroscopic evaluation is sufficient to determine a conclusive cause of death [5-7]. Cardiac conditions are the most frequently identified cause of SUD, with the most common condition being coronary artery disease (CAD). [3] However, in the remaining 15-40% of SUD cases, further investigations are needed due to inconclusive evaluation and a negative autopsy [3,5-7]. A negative autopsy is an autopsy with structurally and microscopically normal cardiac examination, normal toxicology screen, and no findings suggestive of a cause of death [8].

A wide range of conditions may cause SUD with absent or minimal findings at autopsy. For instance, non-cardiac causes include, but are not limited to, epilepsy and metabolic disorders [9]. Regarding cardiac conditions, structural cardiac diseases and arrhythmogenic cardiac disorders represent a significant proportion of SUD in the young [10]. Structural cardiac diseases are usually evident at autopsy; however, in a non-negligible proportion of SCD, which is around 16% in the general population and 4% in athletes, the autopsy is negative [3,7,9]. Since many of these conditions have an underlying genetic basis, the assumption of a hereditary disease as a cause of SUD is often made. Hence, the “molecular autopsy”, which is the process of applying genetic studies as a part of the forensic evaluation to establish the underlying cause of death, is an effective tool that could be utilized in these cases. Interestingly, the molecular autopsy uncovers the cause of SUD in about one-third of these cases [8,11].

SUD, unfortunately, could be the first presentation of an inherited disease [7]. Based on the hereditary nature of the condition, other members of the family are at risk of having a similar fate. If the cause of death was suspected to be genetic, genetic screening of surviving relatives and identification of at-risk individuals can help prevent the tragic outcome of this condition and provide life-saving measures.

In this narrative review, we aim to illustrate the importance of molecular autopsy in evaluating SUD cases, highlight postmortem genetic testing in cardiac and non-cardiac-related SUD cases, and elaborate on how this knowledge could aid in SUD prevention in surviving at-risk relatives.

## Review

Postmortem genetic testing: the molecular autopsy

An appropriate amount of multiple specimens from the decedent’s body at the time of autopsy must be collected in order to proceed with the genetic investigations (i.e., molecular autopsy). Ideally, the preferred samples for genetic analysis are blood in an EDTA tube and fresh frozen tissue from the heart, liver, or spleen [12-14]. There are some controversies in the literature regarding the use of formalin-fixed and paraffin-embedded (FFPE) tissues for genetic investigations. FFPE tissues are obtained for the purpose of histological and immunohistochemical studies, which make them widely accessible when re-examination is considered [4]. DNA derived from these tissues is reported to be unreliable and prone to errors due to alterations in the DNA during the fixation process [15,16]. However, with advanced sequencing technologies, DNA derived from FFPE tissues may be considered an alternative for genetic evaluation if handled with caution to avoid false results [10].

According to the Heart Rhythm Society/European Heart Rhythm Association (HRS/EHRA), in the case of SUD with obscure autopsy findings, performing a comprehensive or targeted four-gene testing for channelopathies may be considered to establish the likely cause of death and subsequently identify at-risk relatives. Targeted genetic testing includes the three genes linked to long QT syndrome (LQTS), namely KCNQ1, KCNH2, and SCN5A, the latter also causing Brugada syndrome (BrS), in addition to the RYR2 gene, which is linked to catecholaminergic polymorphic ventricular tachycardia (CPVT) [12].

Sanger Sequencing

In its early years, DNA analysis relied on Sanger sequencing, which represents a first-generation sequencing technology to test for a few defined genes associated with channelopathies [10]. Sanger sequencing relies on a DNA polymerase reaction that synthesizes numerous copies of the DNA region of interest, using single-stranded DNA as a template [10]. However, with a large number of SUD-related genes identified recently, relying on Sanger sequencing would be time-consuming. In addition, the process of formalin-fixation results in DNA shearing into fragments of an average of 150 base pairs in length, which cannot be used with Sanger assays that require longer fragments with a read length of more than 250 base pairs and high-quality DNA [17]. Even though it is accurate and easy to perform, the high cost per sample, slow speed, limited scale of genes analyzed, and sequencing of a single fragment of DNA at a time are major limitations of this technology [10].

Next-Generation Sequencing

To overcome the limitations of first-generation sequencing technology, a more advanced sequencing technology known as next-generation sequencing (NGS), has been developed. Over recent years, genetic alterations associated with SUD have been increasingly identified. Being very fast and cost-effective for large-scale genetic analysis, NGS allows us to perform a broader targeted gene panel testing, or a more comprehensive genomic study, whole exome sequencing (WES) [10]. WES results in the identification of a great number of variants that undergo further processes of filtration and prioritization to determine their clinical significance and interpretation in relation to SUD. When there is a suspicion of a specific condition or group of diseases, performing targeted gene panel testing is preferred, whereas wider gene panel testing and WES may be preferable in uncertain conditions [10]. Similar to genetic cardiac conditions, genetic non-cardiac conditions may also present with SUD. Thus, with the use of WES, the panel of genes of interest is widely expanded to cover multiple possibilities without having to repeat the sequencing process [11]. Despite the advantages of NGS, Sanger sequencing continues to play an essential role in clinical genomics, which serves as an alternative technique for validating the sequence variants identified by NGS [18].

Although WES is a fascinating technology, it is challenging to assess and identify the variants’ pathogenicity due to the massive number of variants it detects. A large number of these variants are of undetermined significance (VUS). This results in a huge dilemma in the current genetic investigations, as there is insufficient evidence regarding their pathogenicity. If such variants are misinterpreted, it will lead to inaccurate diagnosis, unnecessary treatment, and a profound psychological impact on affected individuals [19]. To overcome this barrier, Schön et al. performed a Human Phenotype Ontology (HPO)-based assessment of WES in 16 cases of SUD with obscure autopsy findings [11]. WES identified an average of 68,947 variants per sample, and after the initial filtration process, an average of 276 variants per sample remained. The next filtration process was mainly based on an HPO-derived “virtual gene panel” created using the following HPO terms: sudden cardiac death, arrhythmia, status epilepticus, and apnea. The advantage of this approach is that newly recognized genes are automatically incorporated into the HPO database and are linked to their related HPO term. Using the HPO‐based gene panel, 1.4% of the variants were prioritized. After the final variant interpretation, 11 potentially causative variants were identified, three of which had not been previously identified [11].

The Use of miRNAs

Since its discovery in the biomedical field in 1993, microRNA (miRNA) analysis has attracted the interest of several researchers. However, the forensic application of miRNA analysis has only been proposed in the last 10 years and is currently receiving great attention. Body fluid identification has been the main goal of the forensic use of miRNA analysis in order to provide confirming universal analysis of unidentified biological stains received from crime scenes or evidence objects. However, there are other forensic uses for miRNA profiling that have demonstrated value but are mostly unexplored and warrant more research, including the identification of organ tissue and the estimation of donor age [20]. According to some studies, miRNAs are very biostable and readily circulate in mammalian blood. They can also be identified in human plasma and serum with a high degree of sensitivity and specificity. Therefore, it was necessary to investigate the diagnostic potential of miRNA detection in human plasma for cardiovascular diseases. The primary cause of SCD is CAD. According to studies, miR-1 and miR-208a levels in the blood considerably increased in acute myocardial infarction (AMI) patients compared to non-AMI controls, suggesting that they might act as biomarkers for the condition. Patients with atherosclerotic cardiovascular disorders have considerably higher levels of miR-135a and lower levels of miR-147 in their plasma, and the ratio of miR-135a/miR-147 might be utilized to diagnose these conditions [21].

Postmortem genetic testing for the evaluation of SUD is recommended in standard guidelines. However, it is not routinely performed, perhaps due to the unavailability of specialist personnel, the lack of equipment in many forensic institutions, and the high cost associated with the procedure [4]. Surprisingly, even with the most advanced genetic technologies, the cause of death may remain undetermined in some cases [10].

Genetic findings in certain specific conditions

The etiologies of SUD in young individuals are extremely diverse. However, in autopsy cases with obscure findings, genetic cardiac diseases are the most probable etiology [5]. These genetic cardiac diseases include channelopathies and cardiomyopathies. Other non-cardiac conditions can cause SUD as well, such as sudden unexpected death in epilepsy (SUDEP) and metabolic disorders.

In this section of the review, the causes of SUD are further discussed and explained.

Postmortem Genetic Testing in Cardiac-Related Causes

Cardiovascular diseases are responsible for about 90% of all sudden death cases in most developed countries [4]. It includes coronary and non-coronary causes, with coronary causes accounting for the majority of cases. CAD mortality rates increase after the age of 40 years and most of them are atherosclerosis-related. The non-atherosclerotic causes include embolism, dissecting aneurysms, arteritis, and congenital abnormalities, among others. Congenital heart defects, hypertensive heart diseases, disorders of the heart valves, myocarditis, and other disorders are considered non-coronary causes [4]. If, however, the initial forensic evaluation does not explain the cause of death, the possibility of genetic cardiac diseases, like channelopathies and cardiomyopathies, is considered, where postmortem genetic testing becomes key to revealing the cause of death [5]. The channelopathies category includes LQTS, short QT syndrome (SQTS), CPVT, and BrS. The cardiomyopathy category includes hypertrophic cardiomyopathy (HCM), dilated cardiomyopathy, arrhythmogenic cardiomyopathy (ACM), restrictive cardiomyopathy, and specific and unclassified cardiomyopathy [2,7].

Dewar et al. conducted a study to investigate the genetic etiologies in obscure autopsy SUD cases using molecular autopsy [22]. The study sample included 191 children who were under the age of five years. The investigators found 11 potentially pathogenic mutations in 12 of the cases, which may have contributed to the diagnosis of 6.3% of obscure autopsy cases in SUD in children. The investigators recommended that channelopathies and cardiomyopathies genes should be included in the genetic testing of SUD in young age groups [22].

Channelopathies are disorders caused by genetic mutations in the genes encoding for cardiac ion channels or the proteins associated with these channels. In cardiac channelopathies, there are no anatomic abnormalities in the heart; instead, there are electrical abnormalities such as ventricular fibrillation or polymorphic ventricular tachycardia leading to sudden death [23,24]. Cardiovascular channelopathies are thought to be responsible for 10-25% of adult SUD and up to a third of SUD in infants and adolescents [7].

Long QT syndrome (LQTS): LQTS is a term used to describe the inherited ion channel disorder and it does not include the acquired causes of QT prolongation such as heart diseases, drugs, hypokalemia, or stroke, where the term “acquired LQTS” is used. Prolonged repolarization is the hallmark of this syndrome. It can be diagnosed by measuring the heart-rate-corrected QT interval (QTc) of more than or equal to 480 ms or by Schwartz criteria, which include clinical history, family history, and 12-lead ECG [10,24].

There are 17 different genes for LQTS. A mutation in any of these genes will lead to the development of LQTS. The most frequently identified genotypes are LQTS1, LQTS2, and LQTS3, which are caused by a loss of function mutation of KCNQ1, KCNH2, and a gain of function mutation of SCN5A, respectively. LQTS has an autosomal dominant mode of inheritance [10,24]. Each genotype has typical phenotypic features. For example, LQT1 is more common in males aged less than 15 years and triggered by exercise, especially swimming, and emotions. LQT2 is more common in females aged more than 12 years, and events are typically triggered by auditory stimulation and the early postpartum period. LQT3 and BrS are more common among males aged >18 years and events are triggered by sleep and rest; events triggered by fever raise suspicion of BrS. The most important risk factors that indicate that a particular person is at high risk are cardiac arrest, syncope, or a QT interval >500 ms at any time during the follow-up. The determination of a genotype is valuable for confirming a diagnosis, detecting high-risk family members, and tailoring the management plan accordingly [24].

A study done by Tester et al. aimed to investigate LQTS in 49 cases of SUD in the young age group [25]. The authors found 10 cases of LQTS, which represented 20% of the study sample; eight of them were females while only two were males. And based on this, they concluded that females were more likely to carry LQTS gene mutations. The mean age at death was 18 ± 11.8 years. They noticed that five deaths occurred during sleep, two at exertion, one at auditory arousal, and two were deemed unknown. The authors concluded that postmortem molecular autopsy of cardiac channelopathies should be considered in the evaluation of SUD cases with obscure autopsy findings [25].

Marcondes et al. conducted postmortem genetic testing for LQTS in 128 SUD cases [26]. Their postmortem genetic testing was able to provide a possible diagnosis in 27 cases, which represented one-fifth of the cases. A total of 31 mutations were found. The most frequently detected genotype was SCN5A; it was detected 14 times, representing 45% of all variants. It was followed by seven cases of KCNH2 (22%) and then by four cases of KCNQ1 (13%). Similar to the previous article [25], LQTS was more common in females than males. In addition, they found that carrying an LQTS gene mutation was associated with having a positive clinical history (mainly seizures) [26].

Brugada syndrome (BrS): BrS is prevalent in approximately one in every 10,000 individuals, most commonly in South Asian countries, and it appears to have a male predominance (70% of cases are males [24]). The diagnosis of BrS is made when the typical ECG feature of BrS is observed either spontaneously or induced by a drug, specifically sodium channel blockers. The typical ECG feature of BrS includes an ST elevation of 2 mm or more and ST depression occurring in the same lead, in at least one right precordial lead. BrS is caused by a loss of function mutation in SCN5A. However, the pathogenic mutation will only be present in a third of the patients, which will be genotypic-negative, and phenotypic-positive. BrS arrhythmic events are typically triggered by vasovagal stimulation like after eating, during the night, and fever [10,24].

Catecholaminergic polymorphic ventricular tachycardia (CPVT)*:* CPVT occurs most frequently in the pediatric age group of 4-12 years and male sex. It is triggered by exercise, especially swimming, presenting as syncope or cardiac arrest. It is less common than LQTS; however, it is more severe as the postmortem findings of CPVT are nearly as common as LQTS in the autopsy of sudden arrhythmic death syndrome (SADS) cases. The most frequently detected pathogenic mutations are RYR2 and CASQ2. CPVT does not have any findings in resting ECG. After the exclusion of structural cardiac disease, CPVT is diagnosed by inducing premature ventricular contractions (PVCs) in the presence of heart rates of more than 100 bmp during a stress ECG test. The PVCs may convert to polymorphic ventricular tachycardia and occasionally to the classic pathognomonic sign of CPVT, which is bidirectional ventricular tachycardia [10,24].

Lahrouchi et al. investigated 302 cases of SADS [27]. The study aimed to identify the value of postmortem genetic testing in SADS. The investigators found that CPVT and LQTS were the leading causes. They diagnosed 17 cases of CPVT and 11 cases of LQTS, representing 6% and 4% of the sample, respectively [27].

Short QT syndrome (SQTS): SQTS is much rarer than other channelopathies. It is diagnosed by a QTc of 340 ms or less, or a QTc of 360 or less plus one or more of the following additional features: the presence of a gain of function mutation in the genes encoding for potassium channels including KCNQ1, KCNH2, KCNJ2; secondly, the presence of SQTS in the family or SCD in young family members; thirdly, surviving a cardiac arrest without any structural abnormalities in the heart [10].

Hypertrophic cardiomyopathy (HCM)*:* in cardiomyopathies, HCM is one of the most frequent autosomal dominant genetic disorders. It is a major cause of SCD in youths and athletes. It is identified by the ventricular septum being asymmetrically hypertrophied, ventricular wall thickening, increased heart weight, and narrowed ventricular cavity. There are two types of pathogenic mutations implicated with HCM: familial and sporadic.

Hereditary cardiomyopathy is the third most common cause of SCD, accounting for 5.9-6.2% of all SCDs [28]. Because establishing the cause of death in cases of sporadic HCM has remained challenging, it becomes a research focus point in forensic pathology. To date, more than 1600 pathogenic mutations have been found in a minimum of 27 genes, with MYH7 mutations being the most common [28].

Marey et al. assessed 35 patients who died of cardiac arrest and were suspected of having cardiomyopathy on the basis of autopsy or clinical evidence [29]. They discovered 15 causal mutations in 15 individuals based on targeted sequencing: three causal variants within the DSP (desmoplakin) gene, three within TNNT2 (troponin T), three within the LMNA (lamin A/C) gene, two within TNNI3 (troponin I), two within MYH7 (beta myosin heavy chain 7), one within TTR (transthyretin) gene, and lastly one within MYBPC3 (myosin-binding protein C). The findings had a variety of impacts on families, including allowing presymptomatic genetic testing in relatives, initiating early intervention based on the particular contributive gene, determining the uncertain diagnosis of borderline cardiomyopathy, and siblings’ reassurance in de novo mutation cases [29].

Gaertner-Rommel et al. reported a case of a 19-year-old with SCD who was further investigated using a combination of forensic and molecular autopsy techniques. In FHL1 and MYBPC3, which are genes found in cardiac muscles, the molecular autopsy revealed two (possibly) harmful genetic variations. The MYBPC3 variant only demonstrated partial penetrance. The FHL1 mutation revealed a de novo type of mutation. In muscle samples, low levels of FHL1 mRNA and no FHL1 protein were found. Indicating nonsense-mediated mRNA decay and/or damaging of the truncated protein revealing a possible disease mechanism in victims of SCD [30].

Arrhythmogenic cardiomyopathy (ACM) and arrhythmogenic right ventricular cardiomyopathy/dysplasia (ARVC/D):ARVC is a poorly recognized and frequently misdiagnosed disorder of the right ventricle (RV) marked by the substitution of the myocardium into fibroadipose tissue. It is estimated that ARVC affects one out of every 5000 individuals, accounting for nearly 20% of SCDs in individuals below the age of 35 years [31]. According to Mu et al., in a cohort of 86 SUD cases, ARVC represented 10.3% of SCD cases and was the second most common cause of SCD [31]. ACM is inherited in a Mendelian autosomal dominant fashion, with disease-causing mutations discovered in more than 13 genes. Approximately half of the clinically diagnosed cases had putative mutations in genes that encode desmosomal proteins: DSP (desmoplakin), PKP2 (plakophilin-2), DSC2 (desmocollin-2), DSG2 (desmoglein-2), and JUP (junction plakoglobin) [32].

Sato et al. investigated mutations in DSP, DSG2, and PKP2 in 15 cases of sudden death in which the cause of death was unknown at autopsy [33]. DSP mutations were detected in three of the cases. Two of the mutations were previously unknown; one had been found in a patient with ARVC, which was identified clinically [33].

Likewise, in one recent case report published by Leone et al., a molecular autopsy was performed on a 13-year-old boy who suddenly died after physical exertion following cardiopulmonary resuscitation [34]. A sample of the victim’s DNA was tested by using NGS in combination with a postmortem examination. Pathogenic heterozygous c.314del (p.Pro105Leufs7) frameshift mutation in the PKP2 gene was discovered. Plakophilin, which is encoded by the PKP2 gene, has been linked to ACM. A cascade of genetic and clinical screening was introduced to 19 family members in total; 12 more individuals were determined to be genotype-positive, with six of them meeting two or more of the main task force criteria (TFC) used for the clinical standard of diagnosing ARVC [34].

Postmortem Genetic Testing in Non-Cardiac-Related Causes

Epilepsy: SUDEP is one of the main causes of the increase in mortality of epileptic patients and can account for up to 18% of deaths in this population [35,36]. It is defined by Nashef et al. as “a sudden, unexpected, witnessed or unwitnessed, non-traumatic, and non-drowning death in patients with epilepsy with or without evidence for a seizure, and excluding documented status epilepticus, in which postmortem examination does not reveal a structural or toxicologic cause of death” [37]. Factors associated with increased risk for SUDEP include generalized tonic-clonic seizures, history of nocturnal seizures, long-standing history of epilepsy, diagnosis at a young age, male sex, intellectual disability, and age between the 20s and 40s [35].

The exact mechanism of SUDEP is unknown; however, many theories have been proposed to explain the underlying mechanism of SUDEP, mostly pertaining to dysfunction in the respiratory, cardiac, and nervous systems [35,36].

Suppression of the autonomic nervous system control over respiration during seizure episodes can lead to severe hypoxia and increased blood CO_2_ levels. Moreover, this can be worsened by airway obstruction that can occur during sleep in the prone position, which was found to be common in SUDEP cases. In addition, normal arousal response to the increased CO_2_ level might be blunted by the suppressed autonomic nervous system. Several studies indicate the significance of respiratory suppression mediated by the seizure activity in the brain in SUDEP cases [35].

The cardiac conduction system may also be affected by seizure episodes; it can either lead to shortening or prolonging of the QT interval, which is a known pathophysiological mechanism of sudden unexpected death in general, such as in autosomal dominant LQTS [35]. It has been proposed that there are genetic associations between channelopathies and epilepsy since some genes that encode the ion channels in the heart are also present in the brain [35,36]. Several studies have reported variants of many channelopathies genes that were found in cases of SUDEP, such as variants of SNC5A, KCNQ1, KCNH2, and HCN1-4 [35].

Many epileptic syndromes have underlying genetic mutations with increased risk of SUDEP,e.g., Dravet syndrome with a genetic variant in SCN1A, early infantile encephalopathy with a genetic variant of SCN8A [35,36], and familial focal epilepsy associated with DEPDC5 gene [36]. Knowledge about these underlying genetics emphasizes the role of genetic analysis in SUDEP and the potential preventive method for high-risk living relatives [35].

Metabolic disorders:SUDI is a broader phenomenon referring to all sudden unexpected deaths in which the age is between birth and up to 12 months, including sudden infant death syndrome (SIDS) cases [38]. SIDS refers to cases in which the cause of death cannot be determined after a thorough autopsy. Several preventive methods have been successful in decreasing the incidence of SIDS, such as having the baby sleep in the supine position [39].

Metabolic disorders are a group of inherited disorders that can lead to SUDI. The majority of these disorders have an autosomal recessive inheritance fashion, and they are generally asymptomatic in early life. Deficiency in the N-acetylglutamate synthase enzyme can occur when there is a mutation in the NAGS variant leading to hyperammonemia. Fatty acid oxidation disorders are major metabolic disorders that are classified into several types, including deficiency of the following enzymes: very long-chain acyl-CoA dehydrogenase, 3-hydroxy-3-methylglutaryl-CoA lyase, and carnitine palmitoyl transferase II; caused by mutations of these genetic variants ACADVL, HMGCL, and CPT2, respectively [39]. The definitive diagnosis of metabolic disorders in SUD cases needs full postmortem examination and metabolic autopsy, which include not only genetic analysis but also a microscopic examination of the liver and postmortem blood acylcarnitine analysis [38].

Outcomes of postmortem genetic testing

Most of the genetic variations that underlie SUD owing to cardiomyopathies or channelopathies are autosomal dominant and have a 50% chance of inheritance. As a result, understanding the genetic variations implicated in SUD cases is critical, not only to know the cause of death in the deceased but also to decide whether genetic screening for surviving relatives should be undertaken [23].

A full clinical history of the deceased should be obtained, including the assessment of the type and level of activities before death and any symptoms that preceded the event. After this, a detailed autopsy including postmortem genetic testing should be performed [8]. Genetic evaluation of the surviving relatives should only be done if the molecular autopsy identifies a diagnosis. Initial clinical assessment of the family should include detailed personal and family history, physical examination, resting ECG, stress ECG, and echocardiography. In certain cases, cardiac MRI and other tests may also be performed [8].

In a study by Marcondes et al., 24 families underwent screening after an occurrence of SUD in their families [26]. The researchers investigated 148 surviving relatives either by clinical assessment alone (64 out of 148) or by clinical assessment combined with genetic evaluation (84 out of 148). Among the 84 people who underwent genetic evaluation, 42 (50%) were found to be carrying genetic mutations. Furthermore, a definitive diagnosis of LQTS or BrS was established in 18 relatives (18 out of 148), representing 12% of the sample, with most of them being females (13 out of 18). Thus, clinical assessment and genetic evaluation of surviving family members proved to be valuable in detecting many at-risk family members [26].

Ethical and financial implications of postmortem genetic testing

Indications of postmortem genetic testing include scientific research purposes, request of the decedent’s family, or, in many countries, as part of judicial investigations into death, especially in young victims [40,41]. Children are more likely than adults to lack anatomic findings that are typically seen in standard autopsies, which necessitates postmortem genetic testing to ascertain the likely cause of death [42]. In the ethical debate over postmortem genetic testing, concerns regarding informed consent, confidentiality, and deceased information disclosure are not new and are not sufficiently addressed either in the literature or by legal frameworks in any of the countries.

In medical practice, informed consent, which is the act of voluntary agreement between the patient and doctor regarding medical care, is a crucial part before any action is performed and is typically obtained from the competent patient [40]. Consent is also obtained from patients who are alive before contacting their relatives to disclose any relevant information. However, in the context of a patient’s death, informed consent for molecular autopsy is obviously not obtained from the deceased individual [40]. If genetic testing is requested by judicial authorities as part of the medico-legal death investigation (hence called public molecular autopsy in some jurisdictions), then informed consent from the family is not required [40]. If genetic testing is done for research purposes, the recommendations are to obtain informed consent from the next-of-kin [40]. If a molecular autopsy is requested by family members (hence called private autopsy), there is variation regarding who is legally authorized to provide consent, although in many jurisdictions the spouse is first on the list [40].

Confidential information regarding individuals must not be disclosed without their authorization even after an individual’s death. However, given the familial nature of genetic information, sharing positive results with close family members may help in preventing serious harm in living individuals who might carry a similar genetic risk [42]. In such cases, familial disclosure is preferred over any professional obligation to ensure individuals' confidentiality after death [40,42]. Unfortunately, there are no clear guidelines on how professionals should deal with families in this context [41]. A multidisciplinary collaboration between forensic pathologists, geneticists, and genetic counselors among others is very important.

The cost of postmortem genetic testing has been frequently reported as a significant barrier. Currently, most third-party payers and insurance companies do not cover the costs of postmortem genetic testing, and hence family members who are willing to do the test are responsible for paying out of their pocket [18]. It is extremely important to anticipate the future individual risk of sudden death in order to provide early interventions that minimize the future costs of illness. Based on the knowledge provided by genetic testing, this risk could be anticipated.

Counseling families affected by sudden death in the young about the results of the molecular autopsy is undoubtedly challenging. In general, the focus of counseling is to explain the findings to the relatives in a way they can understand the scenario, identify other at-risk relatives, and provide referrals for follow-up as needed [42]. In some cases, a three-generation pedigree must be considered. The recommendations are to refer first-degree relatives of a case of SUD with a detected pathogenic variant to a geneticist with experience in interpreting genetic laboratory results and DNA sequencing, and a clinical specialist with expertise in the specific disease in terms of medical and long-term management [42].

## Conclusions

A molecular autopsy is an essential tool for the forensic evaluation of SUD cases requiring medico-legal death investigation; it helps to reach a genetic diagnosis when routine autopsies are unclear, with the ultimate purpose of determining the cause of death besides collaterally leading to the genetic screening of the deceased individual’s family.
